# Vaginal microbiota and mucosal pharmacokinetics of tenofovir in healthy women using tenofovir and tenofovir/levonorgestrel vaginal rings

**DOI:** 10.1371/journal.pone.0217229

**Published:** 2019-05-20

**Authors:** Andrea Ries Thurman, Jill L. Schwartz, Jacques Ravel, Pawel Gajer, Mark A. Marzinke, Nazita Yousefieh, Sharon M. Anderson, Gustavo F. Doncel

**Affiliations:** 1 CONRAD, Eastern Virginia Medical School, Norfolk, VA, United States of America; 2 CONRAD, Eastern Virginia Medical School, Arlington, VA, United States of America; 3 Institute for Genome Sciences, University of Maryland School of Medicine, Baltimore, MD, United States of America; 4 Johns Hopkins University School of Medicine, Clinical Pharmacology Analytical Laboratory, Baltimore, MD, United States of America; Universita degli Studi di Roma Tor Vergata, ITALY

## Abstract

Recent data support that the vaginal microbiota may alter mucosal pharmacokinetics (PK) of topically delivered microbicides. Our team developed an intravaginal ring (IVR) that delivers tenofovir (TFV) (8–10 mg/day) alone or with levonorgestrel (LNG) (20 ug/day). We evaluated the effect of IVRs on the vaginal microbiota, and describe how the vaginal microbiota impacts mucosal PK of TFV. CONRAD A13-128 was a randomized, placebo controlled phase I study. We randomized 51 women to TFV, TFV/LNG or placebo IVR. We assessed the vaginal microbiota by sequencing the V3-V4 regions of 16S rRNA genes prior to IVR insertion and after approximately 15 days of use. We measured the concentration of TFV in the cervicovaginal (CV) aspirate, and TFV and TFV-diphosphate (TFV-DP) in vaginal tissue at the end of IVR use. The change in relative or absolute abundance of vaginal bacterial phylotypes was similar among active and placebo IVR users (all q values >0.13). TFV concentrations in CV aspirate and vaginal tissue, and TFV-DP concentrations in vaginal tissue were not significantly different among users with community state type (CST) 4 versus those with *Lactobacillus* dominated microbiota (all p values >0.07). The proportions of participants with CV aspirate concentrations of TFV >200,000 ng/mL and those with tissue TFV-DP concentrations >1,000 fmol/mg were similar among women with anaerobe versus *Lactobacillus* dominated microbiota (p = 0.43, 0.95 respectively). There were no significant correlations between the CV aspirate concentration of TFV and the relative abundances of *Gardnerella vaginalis* or *Prevotella* species. Tissue concentrations of TFV-DP did not correlate with any the relative abundances of any species, including *Gardnerella vaginalis*. In conclusion, active IVRs did not differ from the placebo IVR on the effect on the vaginal microbiota. Local TFV and TFV-DP concentrations were high and similar among IVR users with *Lactobacillus* dominated microbiota versus CST IV vaginal microbiota.

**Trial registration:** ClinicalTrials.gov NCT02235662.

## Introduction

Over 35 million people worldwide are infected with human immunodeficiency virus type 1 (HIV-1) [1]. Herpes simplex virus type 2 (HSV-2) is one of the most prevalent sexually transmitted infection (STI) globally and is linked to an increased risk of HIV-1 acquisition and transmission [2, 3]. Almost half of all pregnancies worldwide, estimated to be over 100 million annually, are unintended [4–6]. Products that offer protection against multiple STIs (e.g. HSV-2 and HIV-1), or both STIs and unintended pregnancy, termed multi-purpose prevention technologies (MPTs), are urgently needed to reduce these global health burdens. Women have overwhelmingly indicated they prefer an MPT product over single-indication products [7]. CONRAD and our collaborators developed two MPT intravaginal rings (IVRs) which release tenofovir (TFV) alone (active against HIV-1 and HSV-2) and TFV in combination with levonorgestrel (LNG) (contraceptive) for at least 90 days [8, 9], and performed a phase I study in healthy women [10].

Bacterial vaginosis (BV) is characterized by a dysbiotic vaginal microbiota, lacking protective *Lactobacillus* species. BV is present in approximately 29% of United States women [11, 12], 55% of women in sub-Saharan Africa, and up to 70% of female sex workers in South Africa ][13, 14]. Approximately 85% of women with BV are asymptomatic [11]. Data support that BV is likely a cofactor in HIV-1 acquisition (reviewed in [15]) and therefore, assessment of the vaginal microbiota is part of the safety evaluation of HIV-1 prevention products, particularly topical vaginal products. Furthermore, recent subset analyses of women randomized to topical TFV 1% vaginal gel in the CAPRISA 004 cohort and from the FAME 04 cohort among women using TFV 1% vaginal gel or TFV vaginal film found that women with non-*Lactobacillus* dominated vaginal microbiota had reduced mucosal concentrations of TFV and the active metabolite, TFV-diphosphate (TFV-DP), potentially reducing the efficacy of these single agent, peri-coital, topical microbicides [16, 17]. The effect of the microbiota on TFV pharmacokinetics (PK) when TFV is administered topically, but continuously, via an IVR, has not been investigated. We describe the vaginal microbiota among healthy women, before and after the use of an IVR releasing TFV alone or in combination LNG for approximately 15 days. Given the high prevalence of BV and dysbiotic states in the vaginal microbiota of young African women [13, 14], the target users for these IVRs, the data reported in the manuscript are relevant to further development of MPTs.

## Materials and methods

### Clinical study

CONRAD A13-128 was an outpatient, randomized, partially blinded, placebo-controlled, parallel study conducted at the CONRAD Intramural Clinical Research Center at Eastern Virginia Medical School (Norfolk, VA) and PROFAMILIA (Santo Domingo, Dominican Republic). The study was approved by the Chesapeake Institutional Review Board (#Pro00010196) and Comisión Nacional de Bioética (#036–2014), respectively, and registered with ClinicalTrials.gov (#NCT02235662). The study visits and procedures have been previously published [10] and are summarized in S1 Table. Written informed consent was obtained from all participants prior to any study procedures. Participants were healthy, 18–45 years old, had a body mass index of less than 30 kg/m^2^ and reported no use of exogenous hormones and regular menstrual cycles. All women underwent screening at visit 1, to detect the presence of exclusionary factors (e.g. Nugent score of 7–10 [18], active HSV-2, *Neisseria gonorrhoeae*, *Chlamydia trachomatis*, *Trichomonas vaginalis*, HIV-1, Hepatitis B). We instructed participants to refrain from vaginal intercourse and place nothing in the vagina during IVR use. During the study, development of symptomatic BV was exclusionary, but asymptomatic BV after screening was not exclusionary. At visit 4, in the follicular phase of the menstrual cycle (menstrual cycle day 7 ± 1 day), we obtained a vaginal Copan ESwab (Copan Diagnostics, Murrieta, CA, USA) for microbiota analyses, and then participants initiated IVR use. The IVR was removed approximately 15–18 days after insertion, at visit 7. At that time, we obtained another vaginal Copan ESwab for microbiota analysis, two vaginal biopsies for TFV and TFV-DP concentration measurements, and a CV aspirate for TFV concentration.

### Randomization

We randomized participants in a 2:2:1 allocation ratio (TFV/LNG IVR: TFV IVR: Placebo IVR), stratified by study site, to use 1 of the three IVRs for approximately 15–18 days, as previously described [10]. The participants were not told which IVR they had received and the investigator and laboratory staff were blinded to the extent possible. The laboratories performing the microbiota and the PK analyses did not know what treatment participants received. There were no allocation errors.

### Study product

TFV, TFV/LNG, and placebo IVRs were manufactured under current good manufacturing practices at DPT Labs (San Antonio, TX) using manufacturing processes previously described [8, 9]. The unit TFV dose for the IVRs was designed to be approximately 8–10 mg/day of TFV for 90 days of release, and we previously reported the estimated in vivo release over approximately 15 days in this first-in-woman study [10]

### TFV PK assessment

TFV concentrations in plasma, vaginal tissue close to the IVR in the posterior fornix, vaginal tissue distal to the IVR near the vaginal introitus, and cervico-vaginal fluid (CVF) collected by aspirate and on Dacron swabs were determined via previously described liquid chromatographic-mass spectrometric approaches [19, 20] at the Johns Hopkins University. Quantification of TFV-DP in tissue was conducted using a previously described indirect enzymatic approach [21]. Both TFV and TFV-DP were measured in the same biopsy specimen. All assays were validated in accordance with FDA, Guidance for Industry: Bioanalytical Method Validation recommendations [22]. Assay lower limits of quantification were as follows: CVF (aspirate) TFV, 5 ng/mL; tissue TFV, 0.05 ng/sample; tissue TFV-DP, 50 fmol/sample. Laboratory PK analyses occurred in two phases: the first, which included plasma and CVF testing, took place between March and April 2015. The second phase, which included plasma, tissue, and CVF analyses, was conducted between February and May 2016.

### DNA extraction and vaginal microbiota analysis from vaginal swabs

Copan Eswabs were stored at -80 ⁰C at each clinical site and batch shipped on dry ice to the University of Maryland. The swabs were then thawed on ice, and 300 μl of the Amies transport medium containing vaginal secretions were processed using the MoBio Microbiome kit automated on a Hamilton Microlab STAR robotic platform after a bead-beating step on a Qiagen TissueLyser II (20 Hz for 20 min) in 96 deep well plate. Amplification of the V3-V4 regions of the 16S rRNA gene was performed using a two step-PCR in which the sample specific barcode is added during the second PCR, to maximize target amplification. The first PCR used the short 16S rRNA gene specific primers 319F (ACACTGACGACATGGTTCTACA[0–7]**ACTCCTRCGGGAGGCAGCAG**) and 806R (TACGGTAGCAGAGACTTGGTCT[0–7]**GGACTACHVGGGTWTCTAAT**) where the underlined sequence is the Illumina sequencing primer sequence and [0–7] indicate the presence of an heterogeneous pad sequence to improve sequencing quality [23], for a total of 20 cycles. This first step was followed by 10 cycles with primers H1 (AATGATACGGCGACCACCGAGATCTACACNNNNNNNNACACTGACGACATGGTTCTACA) and H2 (CAAGCAGAAGACGGCATACGAGATNNNNNNNNTACGGTAGCAGAGACTTGGTCT) where NNNNNN indicates a sample specific barcode sequence and the underlined sequence corresponds to the Illumina sequencing primer for priming to the first step amplicon. This second step extends the amplicon with the Illumina required adaptor sequences and the sample specific dual barcode system [23]. Amplicons were visualized on a 2% agarose gel, quantified, pooled in equimolar concentration and purified prior to loading on an Illumina HiSeq 2500 modified to generate 300 bp paired-end reads.

The sequences were de-multiplexed using the dual-barcode strategy, a mapping file linking barcode to samples and split_libraries.py, a QIIME-dependent script [24]. The resulting forward and reverse fastq files were split by sample using the QIIME-dependent script split_sequence_file_on_sample_ids.py, and primer sequences were removed using TagCleaner (version 0.16) [25]. Further processing followed the DADA2 Workflow for Big Data and dada2 (v. 1.5.2) (https://benjjneb.github.io/dada2/bigdata.html, [26]). Forward and reverse reads were each trimmed using lengths of 255 and 225 bp, respectively, filtered to contain no ambiguous bases, minimum quality score of two, and required to contain less than two expected errors based on their quality score. Reads were assembled and chimeras for the combined runs removed as per dada2 protocol. Taxonomic classification was performed using speciateIT [27].

Community state types (CSTs) were assigned as previously described [28]. Estimate of absolute abundance was performed using a qPCR of the 16S rRNA gene as previously described [29] and reported as 16S rRNA gene copies per swab. Estimates of phylotypes absolute abundances were calculated by multiplying the relative abundance of each phylotypes obtained by 16S rRNA gene sequencing with the total bacterial 16S rRNA gene copy numbers. Absolute abundance CSTs (aaCST), specific to this cohort, were assigned by hierarchical clustering of Bray-Curtis distances between samples, and each characterized by the top three most abundant bacterial phylotypes. Finally, the microbiota assessment was conducted between October 2016 and January 2017, with data analyses extending to December 2018

### Statistical analyses for comparing changes in vaginal microbiota between baseline and IVR removal

In this Phase I, first-in-woman clinical study, sample size was based on the study’s primary endpoint of safety and feasibility rather than statistical considerations for the interaction between TFV concentrations and microbiota. Subject level comparison of phylotype relative abundances at visit 7 (IVR removal) versus visit 4 (baseline, pre-IVR insertion) in different treatment groups was modeled using a Bayesian binomial-Laplace model. For each phylotype the model estimated the difference of mean logit differences of phylotype relative abundances at visits 7 and 4 within a treatment group (TFV or TFV/LNG IVRs) versus the placebo IVR group (p value) and corrected for multiple comparisons (q value), using Benjamini and Hochberg false discovery rate method [30]. A q value of < 0.05 and an effect size threshold (gEff) of 0.1 was considered significant.

To quantify the effect of IVR use on absolute abundances of vaginal bacterial phylotypes, the log ratios of the absolute abundances of a given phylotype at visit 7 and visit 4 visits were computed and the means of these log ratios computed within each treatment group. The differences of these means between TFV IVR and placebo IVR and TFV/LNG IVR and placebo IVR were estimated using linear regression models.

To compare the structure of the vaginal microbiota, for every visit pair ratio (visit 7/visit 4), Jensen-Shannon divergences were calculated between the relative abundances data at visit 4 and visit 7. The mean Jensen-Shannon divergence of the TFV IVR and TFV/LNG IVR treatment groups were compared to the placebo IVR group. A similar analysis was performed on absolute abundances using Bray-Curtis dissimilarity measures between visit 4 and visit 7.

CST transitions between IVR insertion and IVR removal were described for each IVR cohort, using paired samples. The sample size was too small to perform rigorous statistical comparisons of these transitions. Bray-Curtis dissimilarity was used to cluster the data into four specific aaCSTs for the study population. aaCST transitions between IVR insertion and IVR removal were described. Significance of differences in CST proportions between visit 4 (IVR insertion) and visit 7 (IVR removal) was estimated using Bayesian binomial model with uniform priors on CST proportions.

### Statistical analyses for describing association between vaginal microbiota and TFV PK at IVR removal (visit 7)

At visit 7, we collected both microbiota samples and PK endpoint samples (see S1 Table), and therefore samples at this visit were used for the association between microbiota and mucosal PK. The PK data exhibited a log-normal distribution, and therefore log 10 transformation was applied to avoid violating the normality assumption. The difference between the mean log10 concentrations of TFV in vaginal tissue and CV aspirate among participants with CST IV microbiota at visit 7 versus participants with a *Lactobacillus* dominated microbiota at visit 7 was compared using either ANOVA analysis for CV aspirate or Bayesian mixed effect linear model with subject random intercept term analysis for vaginal tissue TFV and TFV-DP concentrations, due to the fact that each participant had 2 tissue biopsies performed for TFV and TFV-DP concentrations.

An association between the concentrations of TFV or TFV-DP (in the tissue or CV aspirate) and CST (*Lactobacillus* dominated CSTs versus CST IV) was modeled using a Bayesian logistic regression adaptive spline model [spmrf R package] with *Lactobacillus* dominated CSTs combined versus CST IV incidence as the outcome variable and a spline function of log_10_ TFV as the explanatory variable. The proportions of CST IV and *Lactobacillus* dominated CSTs were considered significantly different from each other in a given range of log_10_ TFV concentrations if in that range the 95% credible region around the spline function modeling the mean probability of *Lactobacillus* dominated CSTs (vs CST IV) did not contain the y = 0.5 horizontal line (delineating equality of *Lactobacillus* dominated CSTs and CST IV proportions).

Finally, to determine if significant correlations existed between the relative abundances of individual phyotypes and PK endpoints, we applied Bayesian adaptive spline regression models implemented in the *spmrf*R package [31]. These models were applied to bacterial phylotypes that had at least 20 data points for TFV in CV aspirate or TFV-DP in vaginal tissue concentrations and phylotype relative abundances.

## Results

### Study population

**Fig 1:**
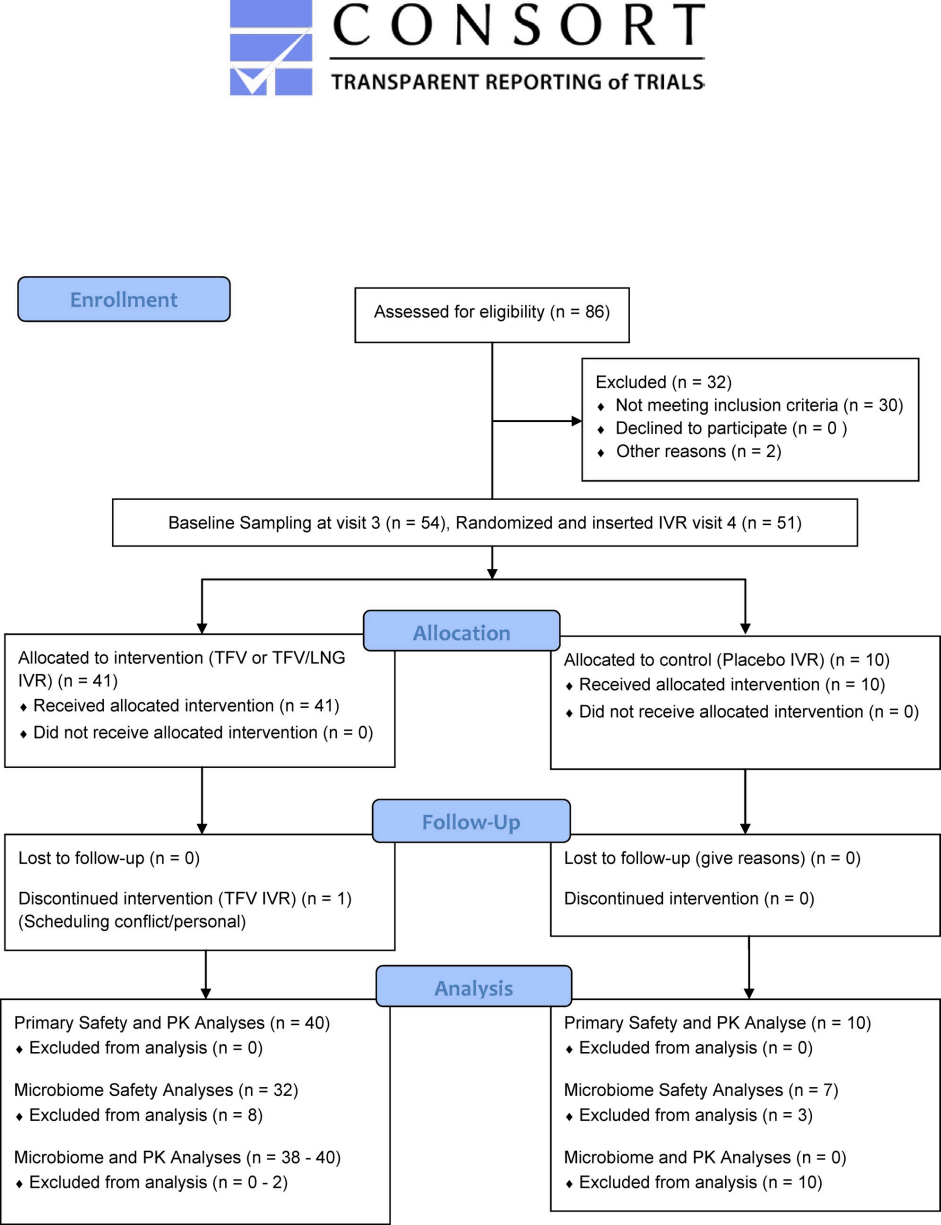
CONSORT Figure CONRAD A13-128 study population.

We enrolled the first patient in October 2014 and the last patient completed the study in December 2015. As summarized in the CONSORT Fig 1, we screened 86 participants, with 51 women initiating IVR use and 50 completing all study visits. The demographic data of the randomized participants were previously published [10] and are summarized in S2 Table. Twenty women were randomized to TFV/LNG IVR, 21 to TFV IVR and 10 to placebo IVR. There were no randomization or allocation errors.

### Effect of IVR use on relative and absolute abundances of vaginal microbiota

There were 101 samples (51 baseline and 50 end of treatment) analyzed for vaginal microbiota. The median total number of individual bacterial RNA sequences was approximately 60,000. After filtering out samples with low sequence counts (less than 2,000 sequences), there were 39 participants with adequate, paired IVR insertion (visit 4) and IVR removal (visit 7) 16S rRNA gene data (Placebo IVR = 7 participants, TFV IVR = 15 participants, TFV/LNG IVR = 17 participants) for the safety analyses.

The mean logit differences between baseline and end of treatment in relative abundances of the five most predominant bacterial phylotypes with the lowest p-values was not significantly different from zero (all q values > 0.895) among TFV, TFV/LNG or placebo IVRs (S3 Table). Thus, the use of the active rings did not significantly modify the composition of the vaginal microbiota. Use of active or placebo IVRs also did not affect the absolute abundances of vaginal bacterial phylotypes. The phylotypes with the lowest p values (all p values < 0.04) for changes in absolute abundances were *Streptococcus*, *Staphylococcus*, *Mycoplasma hominis*, *Dialister propioniifaciens* and *Anaerococcus vaginalis*; however, all q values were > 0.127.

### Effect of IVR use on vaginal bacterial bioburden (estimate of total number of bacteria)

**Fig 2:**
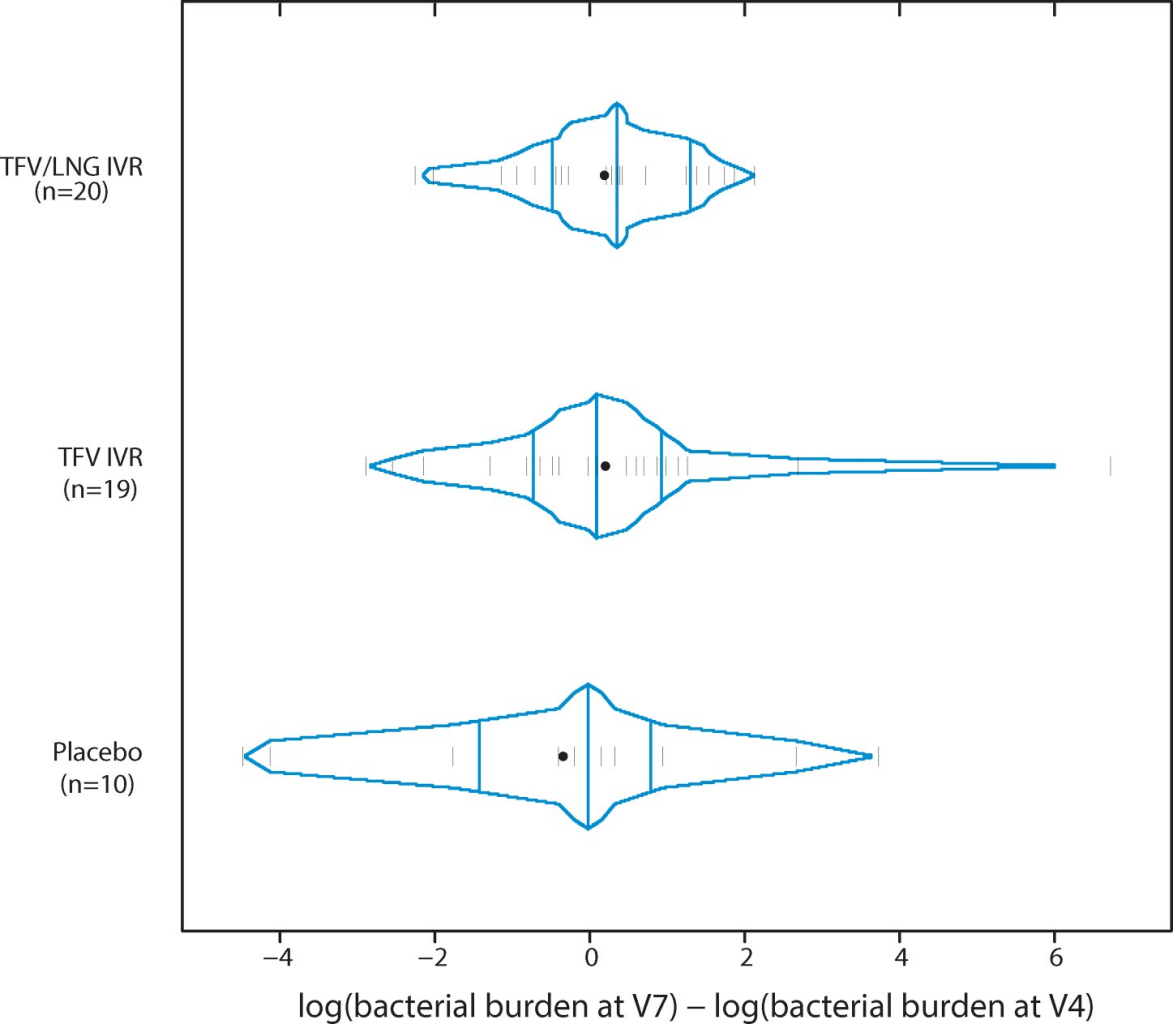
Differences of log absolute bacterial bioburden at visit 7 and visit 4 for TFV, TFV/LNG and placebo IVR users.

Consistent with the data on relative and absolute abundances of vaginal bacterial phylotypes, active IVR use did not change the mean log ratio of absolute bacterial bioburden at visit 7 versus visit 4 (Fig 2) compared to the mean log ratio of absolute bacterial bioburden from placebo IVR use (p = 0.47 for both TFV versus Placebo and TFV/LNG versus Placebo).

### Effect of IVR use on the structure of the vaginal microbiota

**Fig 3:**
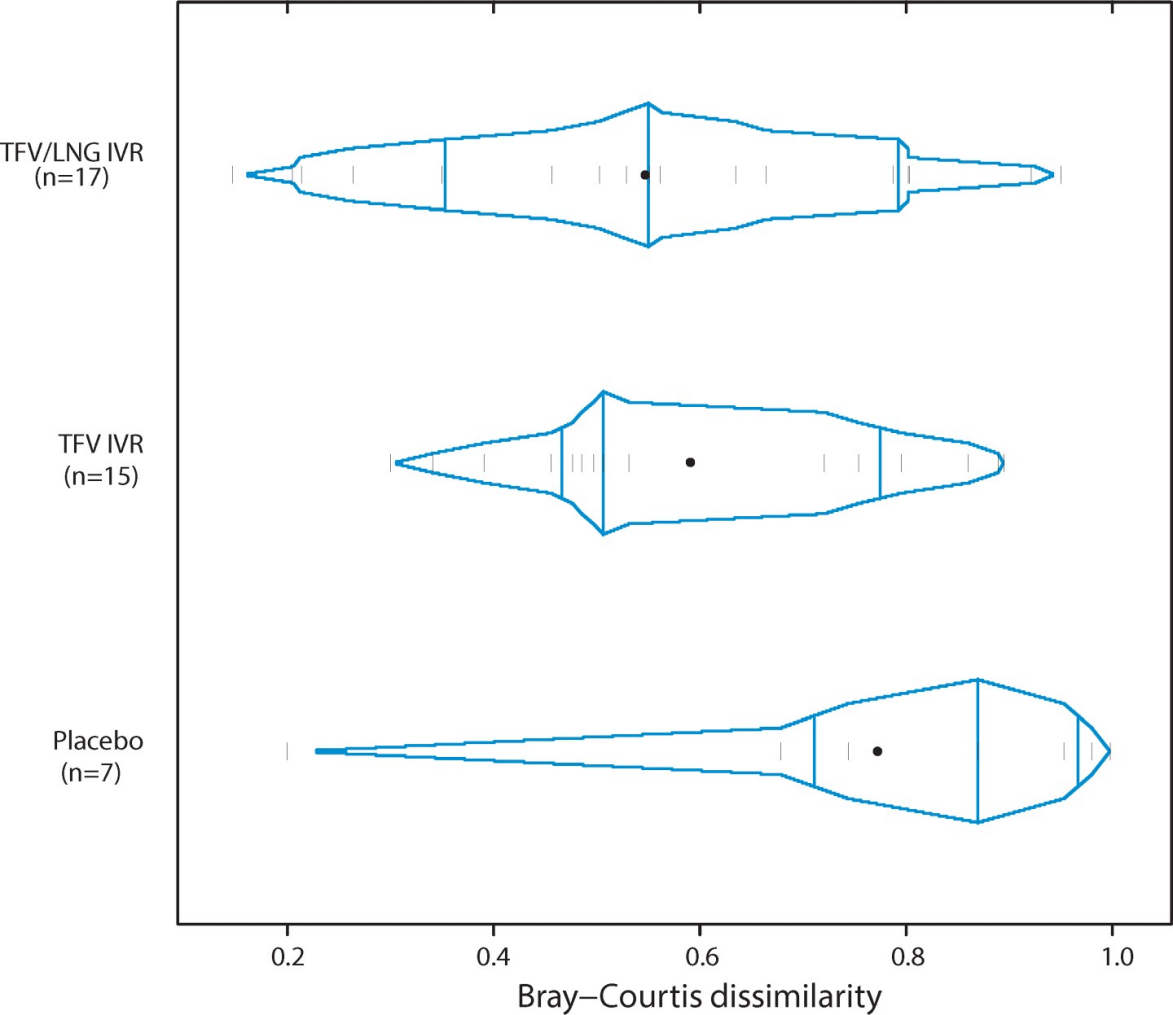
Bray-Curtis dissimilarities calculated using phylotype absolute abundances at visit 7 and visit 4 for TFV/LNG IVR, TFV IVR and placebo user groups.

There were no differences in the structure of the vaginal microbiota using phylotype relative abundances estimated by Jensen Shannon divergences between baseline pre-insertion (visit 4) and end of treatment (visit 7) in active IVR use (TFV p value = 0.28 or TFV/LNG p value = 0.30) compared to placebo IVR use. Similarly, no differences were observed in the structure of the vaginal microbiota using phylotype absolute abundances and estimated by Bray-Curtis dissimilarity between visit 4 and visit 7, in TFV IVR use (δ = -0.18, p value = 0.10) compared to placebo IVR use (Fig 3), indicating that the structure of the community did not change after TFV IVR use. A significant difference was observed in TFV/LNG IVR users compared to placebo IVR users (δ = -0.225, p value = 0.0449) with TFV/LNG users showing more *Lactobacillus*-dominated communities. However, this was the only significant difference found between active and placebo IVR users, which after adjusting for multiple testing, was no longer significant (q-value = 0.0898).

### Effect of IVR use on CSTs

CST transition after IVR use was also evaluated. However, due to the small sample size, rigorous statistical analysis of differences between CST profiles at visit 4 and visit 7, stratified by IVR, could not be performed. The number of participants, for each IVR, with each CST at IVR insertion versus IVR removal, is outlined in frequency Table 1. The majority of IVR users (28/39, 71.7%) (dark highlighted cells, Table 1) did not change CST groups during IVR use, from pre-insertion baseline to end of treatment. Although BV was exclusionary at screening and development of symptomatic BV during the study was exclusionary, a total of 13 women had vaginal microbiota lacking *Lactobacillus* spp, i.e., CST IV, at the time of IVR insertion (visit 4) (Table 1). The majority of these women (n = 8, 4 TFV IVR and 4 TFV/LNG IVR users), maintained their CST IV microbiota at end of treatment (visit 7) (Table 1).

**Table 1:** Frequency table of the number of participants, per IVR, with each community state type (CST) at insertion of IVR at visit 4 (row) versus removal of IVR at visit 7 (column).

Paired Placebo IVR data (n = 7)	CST I	CST II	CST III	CST IV	CST V
CST I	3	0	0	0	0
CST II	0	0	0	0	0
CST III	0	0	1	2	0
CST IV	0	1	0	0	0
CST V	0	0	0	0	0
					
Paired TFV IVR participant data (n = 15)	CST I	CST II	CST III	CST IV	CST V
CST I	5	0	1	0	0
CST II	0	0	0	0	0
CST III	1	0	1	0	0
CST IV	0	0	1	4	1
CST V	0	0	0	0	1
					
Paired TFV/LNG IVR participant data (n = 17)	CST I	CST II	CST III	CST IV	CST V
CST I	6	0	0	0	0
CST II	0	0	0	0	0
CST III	1	0	3	0	1
CST IV	2	0	0	4	0
CST V	0	0	0	0	0

Dark highlighted cells show no change in microbiota between IVR insertion and removal.

Five aaCSTs, specific to this population, were identified. Three of the aaCSTs were dominated by *Lactobacillus* spp. (*L*. *jensenii*, *L*. *iners* and *L*. *crispatus*) and two were dominated by strict anaerobes (BVAB1 and *Atopobium vaginae*). As with the classic CST groupings (28) based on relative abundance, outlined in Table 1, the majority of IVR users (28/39, 71.7%) showed no change in their aaCST with IVR use. Only one placebo IVR user started with a *L*. *iners* dominated microbiota at IVR insertion and transitioned to an *Atopobium vaginae* dominated microbiota at IVR removal. Fifteen women initiated IVR use with either a BVAB1 or an *A*. *vaginae* dominated aaCST. Of these 15 participants, 12 maintained their aaCST with IVR use and three (1 TFV IVR user and 2 TFV/LNG IVR users) transitioned from either *A*. *vaginae* or a BVAB1 dominated aaCST microbiota to a *L*. *iners*, *L*. *jensenii* or *L*. *crispatus* dominated aaCST.

When all samples with adequate bacterial loads were considered, not only paired samples, there were a total of 17 placebo samples, 34 TFV IVR samples and 37 TFV/LNG IVR samples. When this larger sample size was included, no significant differences were found in the proportions of CSTs at visit 4 versus visit 7 for placebo IVR (all p values > 0.14) and TFV IVR users (all p values > 0.52). However, for TFV/LNG IVR users, there were significant increases in the number of participants with CST I microbiota (from 6/17 (35%) at visit 4 to 11/20 (55%) at visit 7 (p < 0.01)); CST II microbiota (from 0% at visit 4 to 1/20 (5%) at visit 7 (p = 0.04); and CST V microbiota (from 0% at visit 4 to 1/20 (5%) at visit 7 (p = 0.05) after treatment. There was also a significant decrease in CST III microbiota (from 5/17 (29%) at visit 4 to 3/20 (15%) at visit 7, p = 0.02) and CST IV microbiota (from 6/17 (35%) at visit 4 to 4/20 (20%) at visit 7, p = 0.02) at end of treatment in TFV/LNG IVR users.

### Effect of the microbiota on mucosal TFV PK

#### CST groupings

The 50 participants using Placebo, TFV or TFV/LNG IVRs were categorized as having either CST IV (n = 11) or *Lactobacillus* dominated microbiota (n = 29) (CSTs I, II, III and V), at end of treatment (visit 7), as shown in Table 2.

**Table 2:** Community state type (CST) frequencies at visit 7 for TFV, TFV/LNG and Placebo IVR users combining all *Lactobacillus* dominated CSTs (CST I, II, III and V), and CST IV.

	CST	Placebo IVR N (%)	TFV IVR N (%)	TFV/LNG IVR (%)	TOTAL ACTIVE IVRs (TFV + TFV/LNG IVRs)(%)	TOTAL ALL IVRs (%)
*Lactobacillus* dominated microbiota	I	5 (50)	6 (30)	11 (55)	17 (42)	22 (44)
	II	1 (10)	0 (0)	1 (5)	1 (3)	2 (4)
	III	1 (10)	5 (25)	3 (15)	8 (20)	9 (18)
	V	0 (0)	2 (10)	1 (5)	3 (8)	3 (6)
CST IV microbiota	IV	3 (30)	7 (35)	4 (20)	11 (27)	14 (28)
Column Totals		10	20	20	40	50

### Association between vaginal CSTs and CV aspirate TFV concentrations

**Fig 4:**
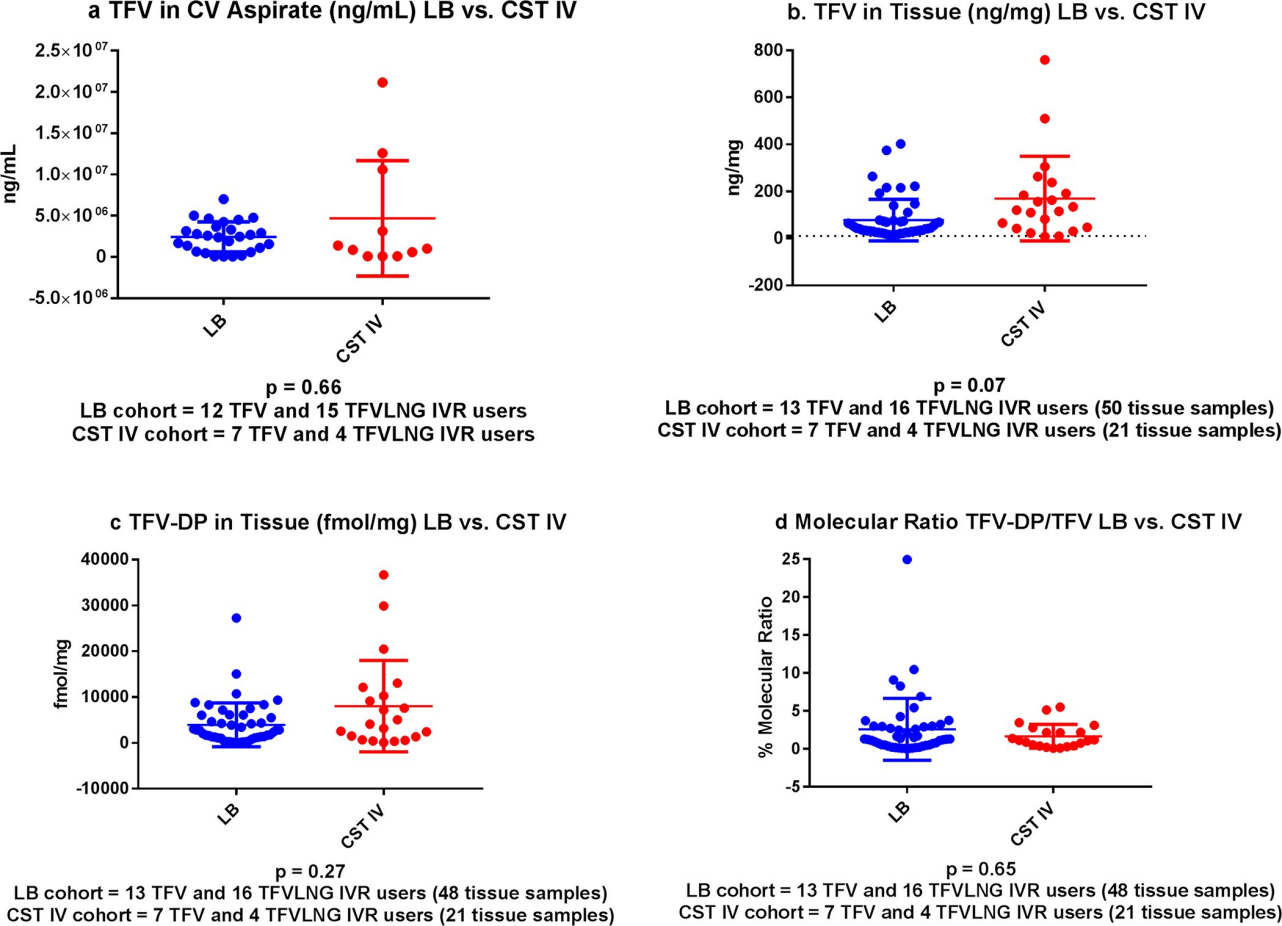
A. TFV concentrations in the CV aspirate among active IVR users with *Lactobacillus* dominated (LB) CSTs versus CST IV. LB cohort (blue dots) includes 12 TFV and 15 TFV/LNG IVR users. CST IV cohort (red dots) includes 7 TFV and 4 TFV/LNG IVR users. B. TFV concentrations in vaginal tissue among active IVR users with *Lactobacillus* dominated CSTs (LB) versus CST IV. LB cohorts (blue dots) includes 13 TFV and 16 TFV/LNG IVR users contributing 50 tissue samples. CST IV cohort (red dots) includes 7 TFV and 4 TFV/LNG IVR users contributing 21 tissue samples. C. TFV-DP concentrations in vaginal tissue among active IVR users with *Lactobacillus* dominated CSTs (LB) versus CST IV. LB cohort (blue dots) includes 13 TFV IVR and 16 TFV/LNG IVR users contributing 48 tissue samples. CST IV cohort (red dots) includes 7 TFV IVR and 4 TFV/LNG IVR users, contributing 21 tissue samples. D. Molecular ratio of TFV-DP to TFV in vaginal tissue among active IVR users with *Lactobacillus* dominated CSTs (LB) versus CST IV. LB cohort (blue dots) included 13 TFV IVR and 16 TFV/LNG IVR users and contributed 48 vaginal tissue samples. CST IV cohort (red dots) included 7 TFV IVR and 4 TFV/LNG IVR users contributing 21 vaginal tissue samples.

**Fig 5:**
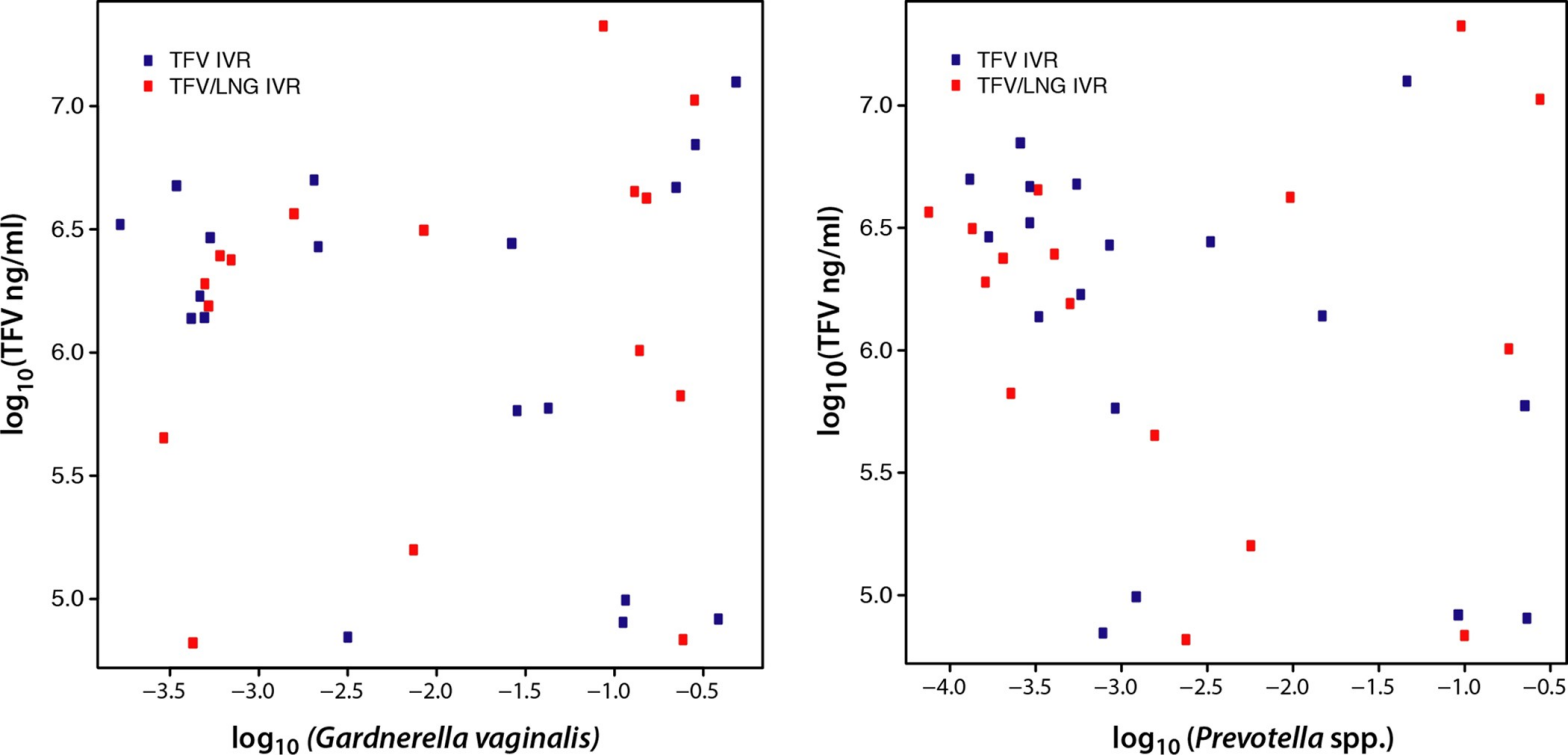
Plot of the log_10_ relative abundances of *G*. *vaginalis* and *Prevotella* spp. and log_10_ of TFV (ng/ml) in CV aspirates at visit 7.

There were 38 participants with evaluable, concurrent TFV CV aspirate and microbiota samples (19 TFV IVR and 19 TFV/LNG IVR users). Among these 38 participants, 12 TFV and 15 TFV/LNG IVR users had *Lactobacillus* dominated microbiota, while 7 TFV and 4 TFV/LNG IVR users had CST IV microbiota. As shown in Table 3, and Fig 4a, there was no significant difference in the mean log TFV concentration (ng/mL) between women with CST IV versus those with *Lactobacillus* dominated CSTs (p = 0.66). Furthermore, the log10 relative abundance of *Garderella vaginalis* or *Prevotella* spp. was not significantly correlated with the log_10_ of the TFV concentration in the CV aspirate (q values 0.47 for both taxa) (Fig 5). The proportion of participants with a CV aspirate TFV concentration of > 200,000 and > 500,000 ng/mL was similar in each cohort (p values 0.23 and 0.43, respectively). All TFV containing IVR users had a CV aspirate concentration of 10,000 ng/mL or higher at the end of treatment (range 0.08–21.2 * 10^6^ ng/mL). Using Bayesian logistic regression model with CST IV versus *Lactobacillus* dominated CSTs as the outcome variables and a spline function of log10 TFV as the predictor variable, we determined that, although CST IV was commonly associated with any dilution degree of TFV, making the overall model non-significant, an enrichment of *Lactobacilli* was found with TFV concentrations in the range of 1.6–6.3 x 10^6^ ng/mL.

**Table 3:** Mucosal concentrations of TFV and TFV-DP based on community state type (CST) at end of treatment (visit 7) for TFV and TFV/LNG IVR users combined.

Variable	CST IV Microbiota				*Lactobacillus spp*. Dominated Microbiota (CST I, II, III, V)				P value
	N	Mean	SD	Median	N	Mean	SD	Median	
Tenofovir									
CV Aspirate (ng/mL) *10^6^	11	4.7	7.0	1.0	27	2.4	1.8	2.5	0.66
Tissue (ng/mg)	21	169.4	180.2	120.7	50	78.0	88.2	41.7	0.07
Tenofovir-Diphosphate									
Tissue (fmol/mg)	21	8,038	9,970	4,098	48	3,949	4,773	2,530	0.27
Molecular Ratio of TFV-DP to TFV (%)	21	1.7	1.6	1.1	48	2.6	4.1	1.3	0.65

### Association between vaginal CSTs and vaginal tissue TFV concentrations

Each IVR user had two vaginal biopsies taken for TFV tissue analyses at visit 7, one proximal to the IVR in the vaginal fornix and one distal to the IVR near the introitus. We previously reported no significant differences in tissue concentrations of TFV and TFV-DP between the proximal and distal tissue biopsies [10]. Evaluable PK (at least one of two tissue samples) and microbiota data were available for 13 TFV and 16 TFV/LNG IVR users, contributing 50 vaginal tissue samples with *Lactobacillus* dominated microbiota. The CST IV cohort included 7 TFV and 4 TFV/LNG IVR users who contributed a total of 21 tissue biopsies. The mean log_10_ TFV vaginal tissue concentration in samples with CST IV microbiota was not significantly different from the mean log_10_ TFV concentration in vaginal tissue from participants with *Lactobacillus* dominated CSTs (p = 0.07) (Table 3, Fig 4B). As with TFV concentrations in the CV aspirate, we modeled the association between TFV tissue concentrations and CST. TFV tissue concentrations ranging from 15.8–75 ng/mg were associated with *Lactobacillus* dominated CST, however the overall model was not significant. Only one participant, randomized to the TFV/LNG IVR, with CST IV, had vaginal tissue TFV concentrations which were lower than 10 ng/mg in both vaginal biopsies. All other participants had vaginal tissue TFV concentrations of 10 ng/mg or higher (range 15–760 ng/mg) in vaginal tissue biopsies.

### Association between vaginal CSTs and vaginal tissue TFV-DP concentrations

Evaluable TFV-DP PK (at least one of two tissue samples) and microbiota data were available for 13 TFV and 16 TFV/LNG IVR users, contributing 48 vaginal tissue samples with *Lactobacillus* dominated CSTs. The CST IV cohort included 7 TFV and 4 TFV/LNG IVR users who contributed a total of 21 vaginal tissue biopsies. There was no significant difference between the mean log_10_ TFV-DP tissue concentrations of participants with CST IV at V7 and the mean log_10_ TFV-DP tissue concentrations of participants with *Lactobacillus* dominated CSTs (p = 0.27) (Table 3, Fig 4C). Table 3 demonstrates that the mean and median TFV-DP tissue concentrations in both cohorts at the end of treatment were higher than 1,000 fmol/mg. There were only 2 participants who had TFV-DP concentrations of less than 1,000 fmol/mg in both their vaginal tissue biopsies, taken near and distal to the IVR: one participant randomized to the TFV/LNG IVR had CST IV and had TFV-DP concentrations of 704 and 338 fmol/mg; the other participant, also randomized to the TFV/LNG IVR had CST III and had TFV-DP concentrations of 141 and 108 fmol/mg. Finally, one participant had only 1 evaluable vaginal tissue biopsy, which had a TFV-DP concentration of 233 fmol/mg; she was randomized to the TFV IVR and had CST I microbiota. The range of TFV-DP tissue concentrations in the remaining samples (37 participants) was 1,028–36,684 fmol/mg.

As with the CV aspirate concentrations, we correlated the log 10 tissue TFV-DP concentrations with 58 individual bacterial phylotypes, to determine if any correlations existed. We found no correlation between tissue TFV-DP concentrations and the relative abundance of any bacterial species (all gEff values larger than -0.10 and or all q values > 0.05) (Table 4). Table 4 demonstrates the 10 phylotypes with the lowest p values and shows the correlation between species and TFV-DP concentrations, corrected for multiple comparisons.

**Table 4:** Effect size and corresponding p and q values for associations between TFV-DP concentration in vaginal tissue and log phylotype relative abundances.

Bacterial Species	gEff	P value	Q value	Median (log10(RA))
Lactobacillus jensenii	-0.0205	0.000193	0.0112	-3.04
Parvimonas micra	-0.0151	0.00324	0.0696	-4.29
Lactobacillus vaginalis	0.0159	0.0036	0.0696	-2.43
Mobiluncus curtisii	-0.0129	0.0123	0.178	-4.54
Prevotella genogroup 1	-0.0139	0.016	0.186	-4.32
Prevotella genogroup 2	-0.0104	0.022	0.213	-3.88
Eggerthella	0.0169	0.0345	0.286	-4.46
Dialister sp. type 2	-0.0099	0.0445	0.323	-4.15
Sneathia sanguinegens	-0.0107	0.061	0.393	-3.77
Lactobacillus gasseri	-0.0090	0.0781	0.453	-3.92

Phylotypes with smallest p values are shown.

### Association between vaginal CSTs and the molecular ratio of vaginal tissue TFV-DP to TFV concentrations

There was no significant difference in the median molecular ratios of vaginal tissue TFV-DP to TFV, expressed as percent conversion (Table 3, Fig 4D). This is a measure of the intracellular efficiency of TFV to TFV-DP cellular conversion.

## Discussion

This is the first study to use 16S rRNA gene sequence analyses to examine the effect of TFV releasing IVRs on the vaginal microbiota composition and structure, and describe the impact of vaginal microbiota composition on topical vaginal TFV PK. These data are important given the current concerns regarding the potential negative impact of the vaginal microbiome on topical antiretroviral PK, with particular emphasis on TFV PK in two recent studies [16, 17] of single agent topical, peri-coitally dosed products.

The effect of the TFV or TFV/LNG IVR was not different than the effect of a placebo IVR on the vaginal microbiota. These conclusions were supported by analysis of the relative and absolute abundances of vaginal bacterial phylotypes and vaginal bacterial bioburden. The majority of IVR users maintained their CST throughout IVR use. Specific aaCSTs, categorized for this population, were also maintained by the majority of IVR users, indicating that IVR use did not affect the structure of the vaginal microbiota. There was no evidence that IVR use increased the incidence of BV and no signals that IVR use caused any detrimental impact on vaginal microbiota. These reassuring safety data are in accordance with previous data, albeit using less sensitive culture and gram stain methods, showing no significant changes in vaginal bacteria among a cohort of healthy, sexually active women using the contraceptive IVR [32–36] for up to 12 months. A pilot study of 6 women with recurrent HSV-2 used 16S rRNA gene pyrosequencing to demonstrate that an acyclovir containing IVR had no adverse impact on vaginal microbiota during 14 days of use [37]. Using mixed methods analyses, we found that the proportion of women with *Lactobacillus* dominated microbiota (CST I, II, III and V) significantly increased and the proportion of women with anaerobe dominated (CST IV) microbiota significantly decreased with TFV/LNG IVR use. Although the sample size was small, these findings support that topical micro-dose LNG does not adversely affect vaginal microbiota. The findings are also in accordance with data from women using the etonogestrel/ethinyl estradiol contraceptive IVR, which showed increases in *Lactobacillus* spp. with IVR use [38].

Our analyses on the effect of the microbiota on TFV PK was based on CST groupings, similar to recent subset analyses of TFV gel [16, 17] and TFV film [16] users. In addition, we examined correlations between individual species which are common in BV associated microbiota and have been associated with subclinical changes in mucosal immunity and inflammation [39], namely *Gardnerella vaginalis* and *Prevotella spp*., and the concentration of TFV in CV aspirate. A recent secondary analysis of women randomized to topical TFV 1% vaginal gel in the CAPRISA 004 cohort [40] found that a non-*Lactobacillus* dominated microbiota (CST IV) reduced the efficacy of TFV 1% vaginal gel in preventing HIV-1, even after adjusting for behavioral and demographic covariates [17], but did not alter the efficacy against HSV-2. TFV concentrations in the CV aspirate were significantly reduced among TFV 1% gel and film users with CST IV microbiota [16, 17]. TFV-DP concentrations in vaginal tissue were also reduced in the film users in the FAME 04 study [16]. This is potentially of concern for episodic, peri-coital, single agent regimens. On the contrary, we found no significant differences in tissue TFV-DP concentrations among IVR users with *Lactobacillus* dominated versus CST IV microbiota. Further, we showed that TFV-DP tissue concentrations did not correlate with any of the most abundant 58 individual bacterial phylotypes.

The effect of the microbiota on mucosal TFV PK was not the primary endpoint in this first in woman phase I study [41], and thus we recognize that our relatively small sample size could introduce a type I error in our conclusions. However, a feasible hypothesis to explain the differences between our findings, i.e., no change in mucosal TFV concentrations with BV associated microbiota, and findings from peri-coitally dosed TFV regimens [16, 17], is that the TFV- containing IVRs release TFV continuously, rather than episodically, at approximately 8–10 mg/day [10]. The continuous, 24 hour, controlled and sustained release of TFV by the IVR, unlike the bolus, immediate release of TFV by the gel or the film, provides sufficient active molecules that penetrate into the CV mucosa and are converted into the active metabolite, TFV-DP [10]. Even if some of the TFV is degraded by bacteria in the lumen of the vagina, as has been modeled in vitro [17], our data support that the continuous release of TFV is enough to maintain protective concentrations of the active metabolite in the tissue inside the target cells.

Although the median TFV concentration in CV aspirates was lower in CST IV bearing women, the difference with those bearing *Lactobacillus* dominated microbiota was not statistically significant. Furthermore, we found that all participants, regardless of their vaginal microbiota, had CV aspirate concentrations of TFV exceeding levels associated with protection against HIV-1 in the CAPRISA 004 cohort [42, 43]. Specifically, a CV aspirate TFV concentration over 100 ng/mL conferred an estimated 65% protection against HIV-1 acquisition, while a CV aspirate TFV concentration of over 1,000 ng/mL provided an estimated 76% protection against HIV-1 [42, 43]. In our study, median TFV concentrations in CV aspirate were 1.0 X 10^6^ ng/mL and 2.5 x 10^6^ ng/mL in women showing CST IV and *lactobacillus* spp. dominated microbiota, respectively.

In addition, although not the primary outcomes of the trials, topical TFV gel reduced acquisition of genital HSV-2 compared to placebo in two previous phase IIb trials [40, 44, 45]. In the CAPRISA 004 cohort, participants with CV aspirate concentrations of TFV of 10,000 ng/mL or higher had a 63% protection against HSV-2 compared to participants with no detectable TFV in CV aspirate [44]. All participants using the active TFV-releasing IVRs in this study had CV aspirate concentrations exceeding 10,000 ng/mL, with a median exceeding 200,000 ng/mL. *In vitro* cell and tissue modeling studies support that the anti-HSV-2 activity of TFV becomes evident at concentrations of approximately 10,000–200,000 ng/mL [46]. Using these data as the benchmark for HSV-2 prevention potential, the TFV containing IVRs delivered adequate levels of TFV whether the user had CST IV or *Lactobacillus* dominated CSTs.

In past studies, tissue concentrations of TFV following the administration of TFV vaginal gel as a single dose, two doses or 14 daily doses, with or without intercourse, were variable ranging from 5.3 ng/mg– 830 ng/mg [21, 41, 47, 48]. High TFV-DP concentrations were correlated with TFV concentrations of at least 10 ng/mg of tissue [21, 47, 48]. Our data support that the TFV and TFV/LNG IVRs deliver sustained high TFV concentrations to the vaginal tissues similar to concentrations seen with TFV gel in phase I PK studies, and should be protective, regardless of vaginal microbiota composition. This is reinforced by tissue levels of TFV’s active metabolite, TFV-DP. The benchmark of 1,000 fmol/mg for TFV-DP levels in tissue comes from PK and efficacy studies of TFV 1% gel in macaques, demonstrating that TFV gel, when applied 30 minutes [49] or even 3 days [50] prior to simian human immunodeficiency virus (SHIV) challenge, protected all or the majority of macaques. Although these benchmarks were based on TFV 1% gel, we found that study participants, regardless of their underlying microbiota, had mean and median TFV-DP tissue concentrations that were 2 to 8 times higher than the 1,000 fmol/mg benchmark.

In accordance with the nature of phase I studies, our healthy cohort was screened to exclude women with STIs, and therefore we do not have data on the effect of these common infections on TFV mucosal PK. In the CAPRISA 004 subset, 22% of participants had an STI diagnosis [17]. Our cohort (n = 50 IVR users, with 41 TFV containing IVR users) was the same size as the cohort (n = 41) of TFV gel or film users evaluated by Hillier et al [16] and was similar in ethnic and racial background. The sample size used in this first-in-woman Phase I study was not powered to detect statistical differences in the microbiota endpoints. Given the inter-individual variability in the results, the small sample size is a limitation. However, unlike the large cohort (n = 688) reported by Klatt et al [17], our study and the FAME 04 sub-analysis [16] provided quantification of TFV-DP concentrations in genital tissue biopsies, which was not feasible in the larger CAPRISA 004 cohort [17]. Larger cohorts are needed in future studies to confirm our findings.

Participants in this first safety trial used the IVRs for only approximately 2 weeks, similar to the brief use (6 days) of topical TFV gel or film previously reported [16]. We instructed participants to refrain from vaginal intercourse during IVR use in this first-in-woman safety study to limit partner exposure to an investigational product. The effect of sexual intercourse on TFV PK in IVR users is not known, but sex has been shown to affect TFV PK in TFV gel users [48].

Although we sampled participants for CV aspirate TFV concentrations several times throughout IVR use [10], we only have concurrent microbiota and PK data at the end of treatment (visit 7) to evaluate. Similarly, we obtained biopsies for tissue levels of TFV and TFV-DP only at IVR removal.

Although a Nugent score of 7–10 was exclusionary at screening and development of symptomatic BV was a reason for discontinuation during the study, we did have a substantial number of participants with asymptomatic CST IV vaginal microbiota at the time of IVR insertion and removal. This enabled us to compare TFV mucosal PK endpoints among women with CST IV versus *Lactobacillus* dominated CSTs (combining CST I, II, III and V). The composition of our aaCSTs was characterized by the same bacterial phylotypes found in a study of African women using the etonogestrel/ethinyl estradiol contraceptive IVR for 3 months, with 93.2%, 57.4% and 37.8% of biofilms from used IVRs contained *Lactobacillus* spp., *G*. *vaginalis* and *A*. *vaginae* respectively [51].

## Conclusions

This first-in-woman study of two IVRs releasing TFV and TFV/LNG showed that the IVRs were well-tolerated and safe [10]. These data support that the IVRs did not adversely change vaginal microbiota composition and structure during 2–3 weeks of use. Importantly, high local levels of TFV and TFV-DP were achieved with the IVRs among women with a diversity of vaginal microbiota, including BV associated bacteria. We propose that these IVRs will fill an important gap as MPTs that women, particularly those in less developed countries, can utilize to protect themselves from HIV-1, HSV-2 and unintended pregnancies.

## Supporting information

S1 Table Study visit overview. (DOCX)

S2 Table Demographics of randomized population. (DOCX)

S3 Table Effect of TFV or TFV/LNG IVR on the relative abundances of vaginal phylotypes compared to the effect of placebo IVR between baseline (visit 4) and after approximately 15 days of IVR use (visit 7). (DOCX)

S1 File CONSORT checklist. (DOC)

S2 File CONRAD protocol A13-128 Version 4. (PDF)
