# Supplementary material for: Vaginal microbiota and mucosal pharmacokinetics of tenofovir in healthy women using tenofovir and tenofovir/levonorgestrel vaginal rings
Source: PLoS One. 2019 May 20;14(5):e0217229. doi: 10.1371/journal.pone.0217229 (PMC6527208; doi:10.1371/journal.pone.0217229)
Supplement: S1 Table — (DOCX) [file pone.0217229.s001.docx]

Supplemental table 1: Study visit overview

|  | Screening/ Enrollment | Pre-Treatment | | IVR in Place | | | | | After IVR Removal | |
| --- | --- | --- | --- | --- | --- | --- | --- | --- | --- | --- |
| Visit Number | V1 | V2 | V3 | V4, Menstrual Cycle Day 6 – 8 | | V5 | V6 | V7 | V8 | V9 |
| Menstrual Cycle or IVR Use Day |  | Menstrual Cycle Day 20 – 22 | Luteal Phase | Pre-Insertion | Post Insertion | 24 hours after V4 | LH surge or day 17 | IVR Removal, 8 - 10 days after V6 | 24 hours post removal | 72 hours post removal |
| Informed Consent, Screening Blood and Genital Specimens | ✓ |  |  |  |  |  |  |  |  |  |
| Confirm Ovulation Serum P4 |  | ✓ |  |  |  |  |  |  |  |  |
| Baseline Samples for Safety, PD |  |  | ✓ | ✓ |  |  |  |  |  |  |
| Swab of Vaginal Microbiome |  |  |  | ✓ |  |  |  | ✓ |  |  |
| Randomize and Initiate IVR |  |  |  | ✓ |  |  |  |  |  |  |
| TFV and LNG PK Samples (Blood, CV Fluid, CM and or Tissue) |  |  |  |  | ✓ | ✓ | ✓ | ✓ | ✓ | ✓ |
| TFV and LNG PD Samples (CV Fluid, CM, CV Tissue) |  |  |  |  |  |  | ✓ | ✓ |  |  |
| Post Treatment Samples for Safety, PD |  |  |  |  |  |  |  | ✓ |  |  |
| Remove IVR |  |  |  |  |  |  |  | ✓ |  |  |
