# Supplementary material for: Vaginal microbiota and mucosal pharmacokinetics of tenofovir in healthy women using tenofovir and tenofovir/levonorgestrel vaginal rings
Source: PLoS One. 2019 May 20;14(5):e0217229. doi: 10.1371/journal.pone.0217229 (PMC6527208; doi:10.1371/journal.pone.0217229)
Supplement: S2 Table — (DOCX) [file pone.0217229.s002.docx]

**Supplemental Table 2:** Demographics of randomized population

|  | TFV/LNG IVR  (n = 20) | TFV IVR  (n = 21) | Placebo IVR  (n = 10) | Total  (n = 51) |
| --- | --- | --- | --- | --- |
| Age (years) | | | | |
| Mean (SD) | 36.0 (4.9) | 33.0 (6.3) | 36.3 (4.8) | 34.8 (5.6) |
| Median | 35.0 | 33.0 | 37.5 | 34.0 |
| Range | (28 - 44) | (23 - 45) | (29 - 44) | (23 - 45) |
| Ethnicity | | | | |
| Hispanic/Latina | 12 | 12 | 6 | 30 |
| Not Hispanic/Latina | 8 | 9 | 4 | 21 |
| Race | | | | |
| Black/African American | 4 | 1 | 2 | 7 |
| White | 4 | 7 | 2 | 13 |
| More than one race | 11 | 12 | 5 | 28 |
| Other | 1 | 1 | 1 | 3 |
| Education (years) | | | | |
| Mean (SD) | 12.5 (3.4) | 12.7 (3.2) | 12.5 (1.4) | 12.6 (3.0) |
| Median | 13.5 | 14.0 | 12.0 | 13.0 |
| Range | (3 - 17) | (7 - 17) | (11 - 15) | (3 - 17) |
| Partner Status | | | | |
| Living with partner | 12 | 15 | 4 | 31 |
| Not living with partner | 4 | 1 | 4 | 9 |
| No partner | 4 | 5 | 2 | 11 |
