# Supplementary material for: Vaginal microbiota and mucosal pharmacokinetics of tenofovir in healthy women using tenofovir and tenofovir/levonorgestrel vaginal rings
Source: PLoS One. 2019 May 20;14(5):e0217229. doi: 10.1371/journal.pone.0217229 (PMC6527208; doi:10.1371/journal.pone.0217229)
Supplement: S3 Table — (DOCX) [file pone.0217229.s003.docx]

**Supplemental Table 3:** Effect of TFV or TFVLNG IVR on the relative abundances of vaginal phylotypes compared to the effect of placebo IVR between baseline (visit 4) and after approximately 15 days of IVR use (visit 7)

| **Species*** | **δ** | **2.5%** | **97.5%** | **P value** | **Q value** |
| --- | --- | --- | --- | --- | --- |
| Effect of TFV IVR Use versus Baseline Compared to Placebo IVR Use versus Baseline | | | | | |
| BVAB3 | -1.97 | -3.42 | -0.143 | 0.0368 | 0.895 |
| Streptococcus | -2.99 | -5.85 | -0.163 | 0.0422 | 0.895 |
| Staphylococcus | -1.73 | -3.46 | 0.00528 | 0.0534 | 0.895 |
| Bacteroides uniformis | -1.58 | -3.02 | 0.0858 | 0.0559 | 0.895 |
| Peptrostreptococcus stomatis | -1.63 | -3.59 | 0.47 | 0.108 | 0.898 |
| Effect of TFV LNG IVR Use versus Baseline Compared to Placebo IVR Use versus Baseline | | | | | |
| Staphylococcus | -1.5 | -3.16 | 0.0961 | 0.0698 | 0.99 |
| Bacteroides uniformis | -1.05 | -2.06 | 0.152 | 0.0838 | 0.99 |
| Anaerococcus vaginalis | -1.99 | -4.72 | 0.604 | 0.115 | 0.99 |
| Streptococcus | -2 | -4.54 | 0.781 | 0.133 | 0.99 |
| Corynebacterium accolens | 0.885 | -0.503 | 2.25 | 0.177 | 0.99 |

* The 5 species with the lowest p values are listed for each comparison
